# Comparison of sexual function after robot-assisted radical prostatectomy and carbon-ion radiotherapy for Japanese prostate cancer patients using propensity score matching

**DOI:** 10.1186/s12885-024-12062-7

**Published:** 2024-03-05

**Authors:** Yoshiyuki Miyazawa, Hidekazu Koike, Daisuke Oka, Hidemasa Kawamura, Nobuteru Kubo, Yuhei Miyasaka, Masahiro Onishi, Takahiro Syuto, Yoshitaka Sekine, Hiroshi Matsui, Tatsuya Ohno, Kazuhiro Suzuki

**Affiliations:** 1 Department of Urology, Gunma University Graduate School of Medicine & Gunma University Hospital, 3-39-22 Showa-Machi, 3718511 Maebashi, Gunma, Japan; 2 Hidaka Hospital, Takasaki, Japan; 3 Department of Radiation Oncology, Gunma University Graduate School of Medicine, Maebashi, Japan; 4 Gunma University Heavy Ion Medical Center, Maebashi, Japan; 5 Syuto Clinic, Maebashi, Japan

**Keywords:** Prostate cancer, Quality of life, Sexual function, Carbon ion therapy, Robot-assisted radical prostatectomy

## Abstract

**Background:**

The quality of life of patients is an important consideration when selecting treatments for localized prostate cancer (PCa). We retrospectively compared sexual function after robot-assisted radical prostatectomy (RARP) and carbon-ion radiotherapy (CIRT) using propensity score matching.

**Methods:**

In total, 127 Japanese PCa patients treated with RARP and 190 treated with CIRT monotherapy were evaluated. We evaluated the Expanded Prostate Cancer Index Composite (EPIC) score before treatment and 12 and 24 months after treatment. After propensity score matching, data from 101 patients from each group were analyzed. The study protocol was approved by the Institutional Review Board of Gunma University Hospital (no. IRB2020-050, 1839).

**Results:**

After propensity score matching, the mean EPIC sexual function summary scores in the RARP and CIRT groups were 46.4 and 48.2, respectively. At 12 and 24 months after treatment, these scores were 27.9 (39.9% decrease) and 28.2 (39.2% decrease) in the RARP group and 41.4 (14.1% decrease) and 41.6 (13.7% decrease) in the CIRT group, respectively. Both groups demonstrated significantly decreased scores after 12 and 24 months of treatment compared to before treatment (all *p* < 0.05). At 12 and 24 months, the sexual function summary score was significantly higher in the CIRT group than in the RARP group (*p* < 0.001).

**Conclusions:**

There was a smaller decrease in the EPIC sexual function score in the CIRT group than in the RARP group. These results provide useful information for treatment decision-making of Japanese PCa patients.

## Introduction

Prostate cancer (PCa) is one of the most common carcinomas worldwide, and its incidence is gradually increasing [1]. Among Japanese males, PCa is the most common cancer and the sixth leading cause of cancer mortality [2]. Treatment recommendations vary depending on the PCa stage at diagnosis. For cases of localized cancer, curative treatment with surgery, radiation, or active surveillance (AS) is recommended. Because of the favorable treatment outcomes for early stage PCa, the risks and complications of treatment should be carefully discussed with patients. A randomized controlled trial conducted in 2023 evaluated the outcomes of localized PCa after AS, surgery, and radiation therapy. Hamdy et al. [3] observed a very low mortality rate at a median follow-up of 15 years among patients with PCa diagnosed based on prostate-specific antigen testing, regardless of AS, prostatectomy, or radiation therapy. Radical treatment of PCa was associated with a lower risk of disease progression compared to AS, although there was no difference in the mortality rate. Most studies of patients with PCa who underwent AS demonstrated that 15–41% required a change in treatment within 5 years of starting surveillance [4], whereas 5–10% of men required active treatment due to anxiety [5]. Patients who underwent external-beam radiation therapy had significantly higher quality-of-life (QOL) scores related to stress compared to patients who underwent AS [6]. These findings demonstrate that physicians should take into account the personality and wishes of patients when making treatment decisions. Carbon-ion radiotherapy (CIRT) is the preferred treatment for localized and locally advanced PCa due to its unique physical and biological advantages. The dose distribution of CIRT is more favorable for PCa compared to external beam irradiation due to its superior dose characteristics [7]. Additionally, carbon-ion beams have a high relative biological effect (RBE) due to their high linear energy transfer, which is almost 3-fold higher than that of photons and protons [8, 9]. Our institution uses robot-assisted radical prostatectomy (RARP) and CIRT to treat localized PCa. Although both treatments are associated with a favorable long-term prognosis, they are associated with post-treatment sequelae. In the present study, we used propensity score matching to adjust for background variables of patients receiving the two treatments and evaluated post-treatment changes in sexual function. Given that few comparative studies have evaluated RARP and CIRT, we believe that our study provides useful information to facilitate appropriate decision-making.

## Patients and methods

### Patients and study design

**Fig. 1:**
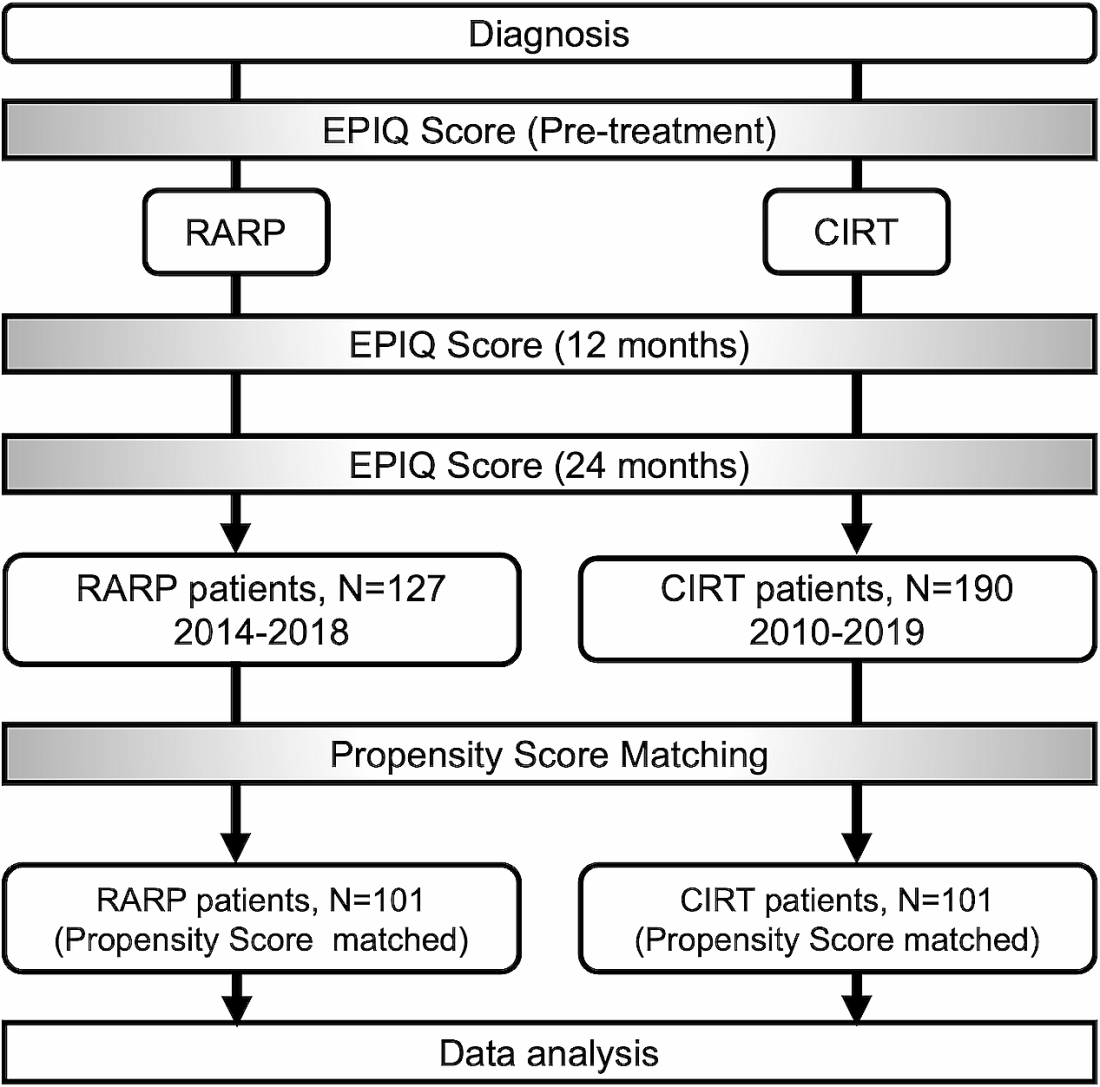
The schema of our research design. This is a schema that shows our research design. EPIC; Expanded Prostate Cancer Index Composite, CIRT; carbon ion radiation therapy, RARP; robotic-assisted radical prostatectomy

Figure 1 presents the study design. In this retrospective study, we compared two cohorts obtained from previous studies, including a cohort of 127 patients who underwent RARP at Gunma University Hospital in Japan between 2014 and 2018, and a comparison cohort comprising 190 patients who underwent CIRT between 2014 and 2018. To evaluate sexual function, we excluded patients who had received androgen deprivation therapy. RARP was performed using the standard transperitoneal approach combined with posterior and lateral approaches using the da Vinci Si system. In cases with cores on only one side, RARP was performed using the nerve-sparing (NS) technique on the unilateral side. Therefore, bilateral NS cases were not included in our study. CIRT was administered at a total dose of 57.6 Gy in 16 fractions over 4 weeks, with a fractional RBE of 3.6 Gy at 4 fractions per week. We evaluated the Expanded Prostate Cancer Index Composite (EPIC) sexual function score before and 12 and 24 months after treatment [10]. Propensity score matching was performed using the age at treatment, prostate-specific antigen value, T stage, and pretreatment EPIC comprehensive score of sexual function as covariates. After one-to-one propensity score matching, we obtained two cohorts of 101 background-adjusted patients in the RARP and CIRT groups.

### Statistical analysis

We used one-to-one propensity score matching, Student’s t-test, Welch t-test, Fisher’s test, and Wilcoxon signed-rank test to examine the association between RARP and CIRT. Two-sided p-values < 0.05 were considered indicative of statistical significance. Comparisons among three groups were conducted using one-way analysis of variance. The mean propensity score at matching was 0.401 ± 0.301, and the caliper was set at 0.08. SPSS software (version 28.0; IBM Corp., Armonk, NY, USA) was used for propensity score matching analyses. Unless otherwise specified, numerical values are expressed as means ± standard deviation.

## Results

### Background characteristics and propensity score matching

Table 1 presents the background characteristics of the participants in the RARP and CIRT groups before and after propensity score matching. Before matching, there were significant differences in PSA and T stage, whereas after adjustment, these characteristics were matched between the two groups. Although there were significant differences in the Gleason score, these differences did not significantly impact sexual function. Therefore, we analyzed sexual function without adjusting for the Gleason score. After adjustment using the propensity score matching method, we analyzed data from 101 patients in each group.

**Table 1:** Characteristics of patients before and after propensity score matching

		Pre PSM			Post PSM		
		RARP, Pre PSM *N* = 127	CIRT, Pre PSM *N* = 190	p-Value	RARP, Post PSM *N* = 101	CIRT, Post PSM *N* = 101	p-Value
Age (mean ± S.D.)		64.9 ± 6.51	65 ± 6.61	*p* = 0.589	65.1 ± 5.87	65.3 ± 6.47	*p* = 0.800
PSA (mean ± S.D.)		7.24 ± 3.40	5.71 ± 1.87	*p* < 0.001	6.34 ± 2.46	6.31 ± 2.07	*p* = 0.939
EPIC Sexual Function summary score (mean ± S.D.)		46.7 ± 15.2	46.9 ± 15.6	*p* = 0.898	46.4 ± 15.6	48.2 ± 16.2	*p* = 0.438
T stage; n, (%)	T1	16 (12.6%)	76 (40.0%)	*p* < 0.001	16 (15.8)	17 (16.8)	*p* = 0.982
	T2	103 (81.0%)	113 (59.5%)		84 (83.2)	83 (82.2)	
	T3	8 (6.3%)	1 (0.5%)		1 (1.0)	1 (1.0)	
Gleason Score; n, (%)	6	8 (6.3%)	49 (25.8%)	*p* < 0.001	7 (6.9)	28 (27.7)	*p* < 0.001
	7	96 (75.6%)	140 (73.7%)		78 (77.2)	72 (71.3)	
	8	17 (13.4%)	1 (0.5%)		12 (11.9)	1 (1.0)	
	9	6 (4.7%)	0 (0%)		4 (4.0)	0 (0)	
Nerve sparing; n, (%)	(+)	99 (78.0%)	NA	NA	76 (75.2%)	NA	NA
	(-)	28 (22.0%)	NA		25 (24.8%)	NA	

RARP; robotic-assisted radical prostatectomy, CIRT; carbon ion radiotherapy, PSM; propensity score matching, PSA; prostate specific antigen, EPIC; expanded prostate cancer index composite, NA; not applicable

### Comparison of EPIC sexual domain scores

**Fig. 2:**
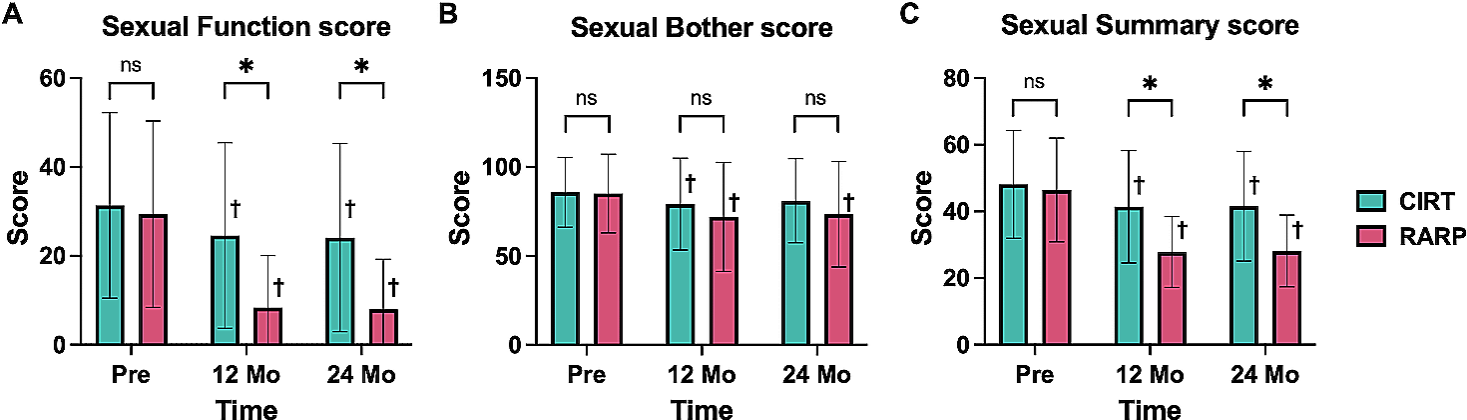
The change of EPIC sexual function score RARP versus CIRT. Comparison of EPIC evaluations between the RARP and CIRT groups at each measurement point. (**A**) Sexual Function score results. (**B**) Sexual Bother score results. (**C**) Sexual Function summary score results. CIRT; carbon ion radiation therapy, RARP; robotic-assisted radical prostatectomy, Mo; month, ns; no significant difference, * *p* < 0.05, CIRT vs. RARP, + *p* < 0.05, vs. Pre-treatment

The two domains of the EPIC sexual score (sexual function and sexual bother scores) and the sexual summary score were compared between the groups. In the CIRT group, the mean sexual function score before treatment (31.4 ± 20.8) decreased by 21.7% at 12 months (24.6 ± 12.0) and by 23.2% at 24 months (24.1 ± 20.5) after treatment. Conversely, in the RARP group, the pretreatment score decreased from 29.4 ± 20.8 to 8.3 ± 12.0 (71.8% decrease) at 12 months and 7.9 ± 10.8 (73.1% decrease) at 24 months after treatment. The CIRT group had significantly higher scores than the RARP group at 12 and 24 months (*p* < 0.001) (Fig. 2A). In both groups, the sexual function scores at 12 and 24 months were significantly lower than the pretreatment score (*p* < 0.001). Furthermore, in the CIRT group, the mean sexual bother score before treatment (85.8 ± 18.9) decreased by 7.7% at 12 months (79.2 ± 26.4) and 5.5% at 24 months (81.1 ± 25.4) after treatment. In the RARP group, the pretreatment score (85.1 ± 21.6) decreased by 15.4% at 12 months (72.0 ± 31.3) and 13.7% at 24 months (73.4 ± 31.1) after treatment. There were no significant differences in scores between the groups at 12 months (*p* = 0.077) or 24 months (*p* = 0.071). In both groups, there was a trend toward a slight decline in the sexual function score. In the RARP group, the sexual bother score was significantly lower at 12 and 24 months after treatment compared to before treatment (*p* < 0.001). In comparison, in the CIRT group, the score was significantly lower at 12 months after treatment (*p* = 0.027) but was not significantly decreased after 24 months (*p* = 0.171) (Fig. 2B). Furthermore, in the CIRT group, the sexual summary score before treatment (48.2 ± 16.2) decreased by 14.1% (41.4 ± 16.9) at 12 months and 13.7% at 24 months (41.6 ± 16.5) after treatment. In the RARP group, the score before treatment (46.4 ± 15.6) decreased by 39.9% at 12 months (27.9 ± 10.6) and 39.2% at 24 months (28.2 ± 10.8) after treatment. The score was significantly higher in the CIRT group than in the RARP group at 12 and 24 months after treatment (*p* < 0.001). In both groups, the sexual function summary scores were significantly lower at 12 and 24 months after treatment than before treatment (*p* < 0.001) (Fig. 2C).

### Influence of pretreatment sexual function scores on posttreatment sexual bother score

**Fig. 3:**
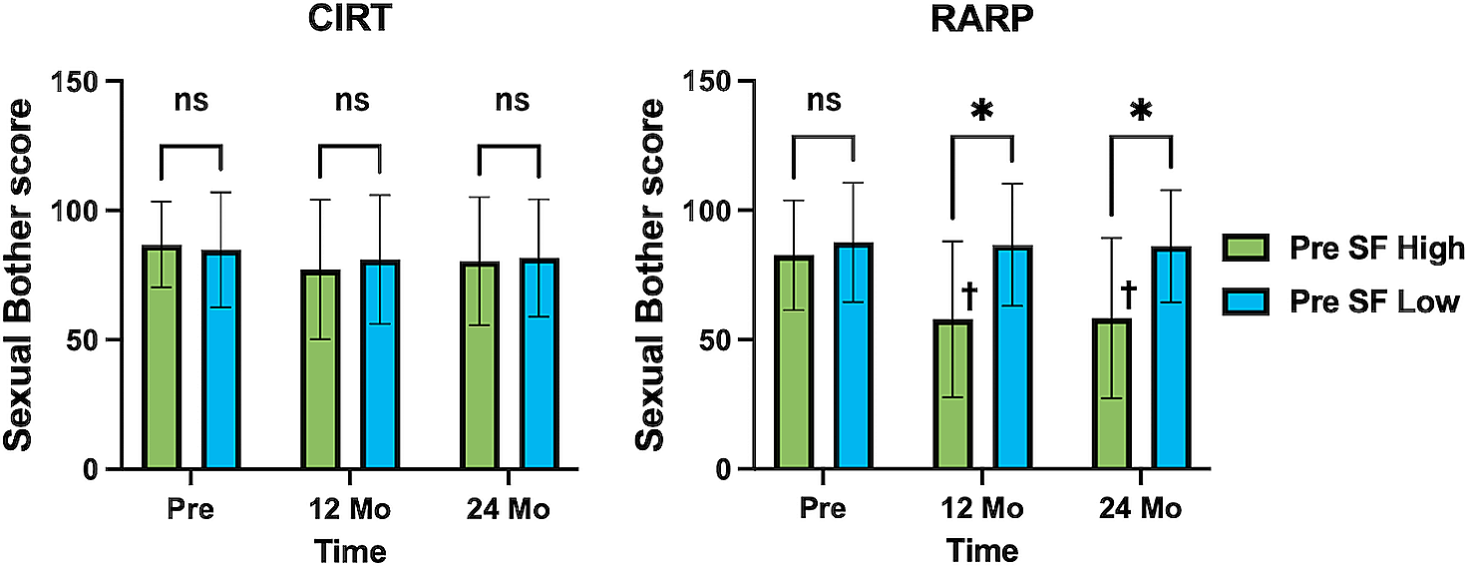
Change in sexual function score grouped by pre-treatment sexual function score. Comparative evaluation of Sexual Bother scores focusing on Sexual Function score before treatment. SF; sexual function score, CIRT; carbon ion radiation therapy, RARP; robotic-assisted radical prostatectomy, Mo; month, ns; no significant difference, * *p* < 0.05, Pre SF High vs. Pre SF Low, + *p* < 0.05, vs. Pre-treatment

Participants from both the RARP and CIRT groups were categorized into the high and low SF groups based on the median sexual function score. The sexual bother scores were compared between these groups. Among patients who underwent CIRT, there were no significant differences in the sexual bother scores between the high and low SF groups at 12 or 24 months after treatment (*p* = 0.610 and = 0.469, respectively). However, among patients who underwent RARP, the high SF group demonstrated significantly lower sexual bother scores compared to the low SF group at both 12 and 24 months after treatment (both *p* < 0.001). In the high SF group that underwent RARP, sexual bother score at 12 and 24 months after treatment was significantly worse compared to the pretreatment levels (both *p* < 0.001) (Fig. 3).

### Influence of NS on sexual function

**Fig. 4:**
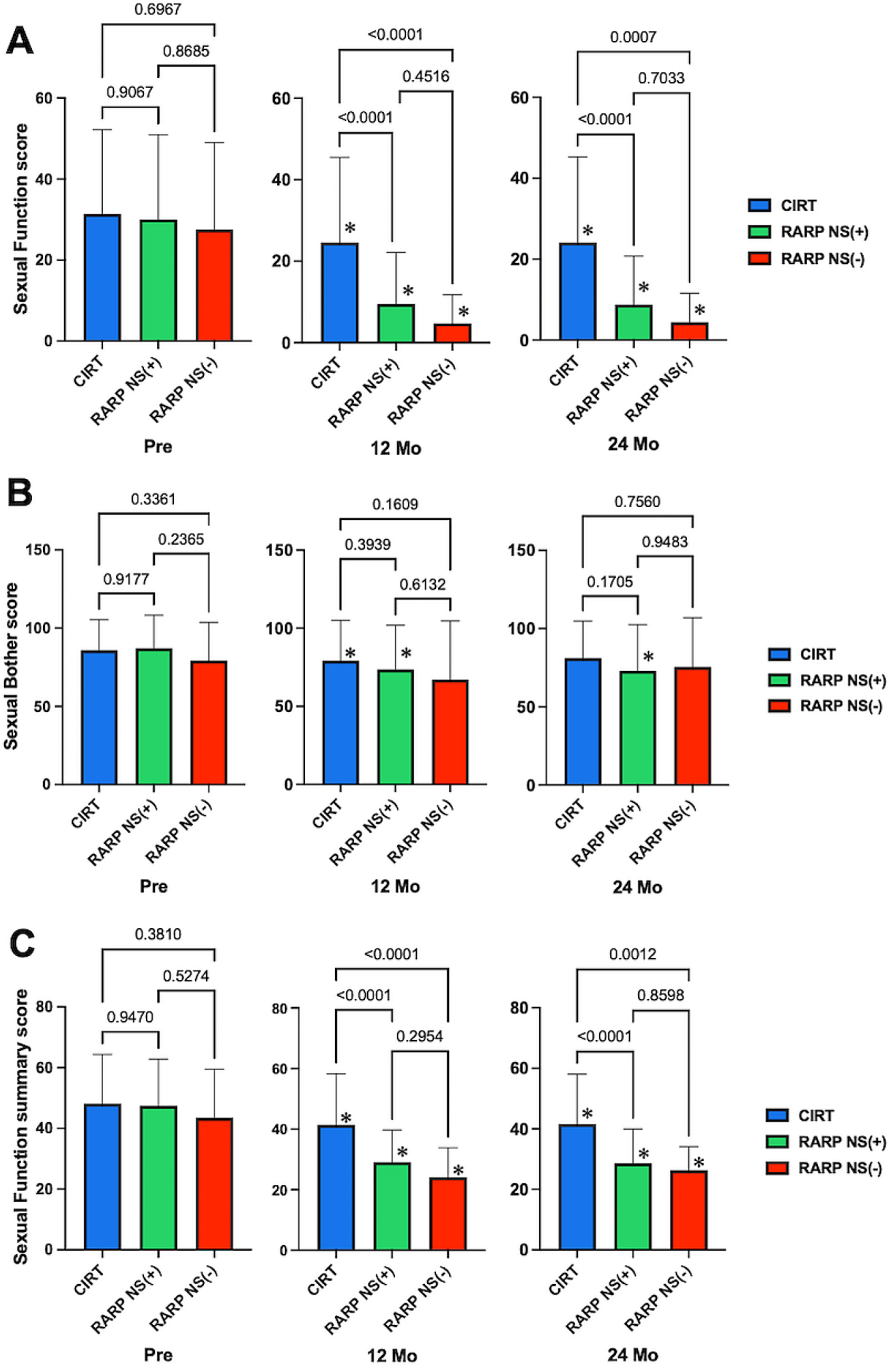
Effect of nerve-sparing in RARP on EPIC sexual function score. Comparison of EPIC evaluations between the RARP NS (+), RARP NS (-), and CIRT groups at each measurement point. (**A**) Sexual Function score results. (**B**) Sexual Bother score results. (**C**) Sexual Function summary score results. CIRT; carbon ion radiation therapy, RARP; robotic-assisted radical prostatectomy, NS; nerve-sparing, Mo; month, Bars, and numbers indicate p-values for each group comparison, * vs. *p* < 0.05, vs. Pre-treatment

We examined sexual function (score: 76) in 101 participants (75.2%) who underwent RARP using the NS strategy. The mean sexual function scores for the RARP NS(+) group decreased from 30.0 ± 20.9 before treatment to 9.47 ± 12.7 (68.4% decrease) at 12 months and 8.78 ± 12.0 (70.7% decrease) at 24 months after treatment. In the RARP NS (−) group, the score decreased from 27.6 ± 21.4 before treatment to 4.70 ± 7.04 (83.0% decrease) at 12 months and 4.41 ± 7.16 (84.0% decrease) at 24 months after treatment. At 12 and 24 months after treatment, the score was significantly higher in the CIRT group than in the RARP NS (+) and RARP NS (−) groups (*p* < 0.001). At 12 and 24 months after treatment, the RARP NS (+) group showed a trend toward significantly higher scores compared to the RARP NS (−) group, although the difference was not statistically significant (*p* = 0.451 and = 0.703, respectively) (Fig. 4A). The mean sexual bother scores for the RARP NS (+) group decreased from 87.1 ± 21.2 before treatment to 73.5 ± 72.9 (15.6% decrease) at 12 months and 73.0 ± 29.5 (16.2% decrease) at 24 months after treatment. In the RARP NS (−) group, the score decreased from 79.3 ± 24.5 before treatment to 67.1 ± 37.5 (15.4% decrease) at 12 months and 75.5 ± 31.4 (4.8% decrease) at 24 months after treatment. There were no significant differences among the three groups at 12 or 24 months (all *p* > 0.05) (Fig. 4B). In the RARP NS (+) group, the mean sexual function summary score before treatment (47.4 ± 15.4) decreased by 38.8% at 12 months (29.0 ± 10.7) and 39.7% at 24 months (28.6 ± 11.4) after treatment. In the RARP NS (−) group, the pretreatment score (43.5 ± 16.0) decreased by 44.6% at 12 months (24.1 ± 9.75) and 39.5% at 24 months (26.3 ± 7.85) after treatment. At both 12 and 24 months, the scores were significantly higher in the CIRT group than in the RARP NS (+) and RARP NS (−) groups (*p* < 0.01). There was a trend toward higher scores in the RARP NS (+) group than in the RARP NS (−) group at both 12 and 24 months, although the difference was not statistically significant (*p* = 0.295 and = 0.860, respectively) (Fig. 4C).

## Discussion

The main treatment options for localized PCa are surgery and radiation therapy. Although open surgery was commonly performed in the past, the widespread adoption of laparoscopic surgery followed by RARP has increasingly established it as the standard treatment worldwide. Furthermore, advancements in radiation therapy have prompted a shift from conventional external beam radiation therapy to particle beam therapy, such as proton and CIRT. Our institution has accumulated experience with CIRT [11]. When managing patients with localized PCa who select curative treatment, most physicians discuss the advantages and disadvantages of both surgical resection and radiation therapy. For patients predicted to have equally favorable outcomes with both treatments, the risk of complications is the main factor determining treatment selection. Few studies have compared sexual function after various treatments. To the best of our knowledge, this study is the first to compare self-reported outcomes related to sexual function among patients who underwent RARP and CIRT. The randomized controlled PROTECT trial compared sexual function in terms of EPIC scores among patients who underwent radical prostatectomy and radiation therapy [3, 12]. In this trial, patients were categorized into AS, radiation therapy, and radical prostatectomy groups. The results demonstrated that the sexual summary score decreased by approximately 50% at 12 and 24 months in the radical prostatectomy group, and by around 70% in the radiation therapy group. Our findings suggest that the radiation therapy group had more favorable outcomes than the radical prostatectomy group. Additionally, the sexual bother score exhibited similar changes to the sexual summary score [12], in line with the functional decline and perceived distress.

Interestingly, the PROTECT trial demonstrated significant decreases in sexual function and sexual bother scores among Western patients. Conversely, we found that the sexual bother score was not significantly different before treatment compared to 12 and 24 months after treatment. Wakatsuki et al. [13] evaluated the QOL of 150 Japanese low-risk PCa patients who received CIRT without additional ADT using the University of California Los Angeles Prostate Cancer Index and patient-reported outcomes. The sexual function score decreased by approximately 21%, from 38.9 before treatment to 30.7 at 12 months after treatment. However, the sexual bother score was not significantly different, being 72.9 before treatment and 71.8 at 12 months after treatment. Similar results were obtained in our study. Namiki et al. [14] compared pre- and post-treatment sexual function among Japanese and American patients who had undergone radical prostatectomy. The pretreatment sexual function was significantly worse in Japanese patients compared to American patients. However, a significant trend toward recovery was observed in the American group compared to the Japanese group. Although both groups had similar sexual bother scores before treatment, the American group showed a decline for up to 6 months after treatment, followed by a trend toward recovery up to 24 months. However, no recovery was observed in the Japanese group. Namiki et al. [14] conducted further investigations to determine the influence of race and culture on sexual function among 352 Caucasians living in the United States, 54 Japanese Americans living in the United States, and 412 Japanese men living in Japan. The sexual bother score before PCa was significantly worse in Japanese men than in Caucasians and Japanese Americans. Furthermore, Japanese Americans with a Japanese American partner had a significantly higher sexual bother score [15]. These results suggest that the sexual bother score is affected by race. However, non-biological environmental factors, such as the living environment, including spouses, might also influence these scores. In our study, the sexual bother score was significantly reduced after treatment in the RARP and CIRT groups, suggesting an influences of lifestyle and behavioral factors on Japanese and Western populations. In this study, we used the Japanese version of EPIC, which was translated from the original version of EPIC after repeated discussions regarding the interpretation of questions related to sexual bother. Therefore, it is essential to consider the influence of cultural background when interpreting the results of QOL questionnaires [10].

Several strategies have been developed to mitigate the sexual dysfunction associated with treatment. Multiple studies have demonstrated significantly better outcomes following RARP in terms of erectile function and sexual function when the NS strategy was used [16, 17]. A meta-analysis revealed that urinary continence was significantly improved with Retzius-sparing RARP than with conventional RARP [18, 19]. However, there were no significant differences in post-treatment sexual function. In our study, there was a statistically non-significant trend toward higher sexual function scores in patients who underwent RARP without NS compared to those with NS. Furthermore, the insertion of a hydrogel spacer around the prostate before radiation therapy reduces adverse events, particularly those related to the rectum [20] and sexual function [21]. Therefore, the use of a hydrogel spacer should be investigated further.

In the present study, patients treated with RARP who had high pre-treatment sexual function scores experienced a significant increase in sexual bother scores compared to those with low pre-treatment sexual function scores. Conversely, among patients who underwent CIRT, the sexual bother score was not significantly different between those with high versus low pre-treatment sexual function scores. CIRT is an effective treatment option for patients with good pre-treatment sexual function to prevent post-treatment worsening of sexual bother.

Our study had several limitations. First, the RARP cohort we examined did not include cases with bilateral NS. It has been reported that cases with bilateral NS had significantly better post-treatment sexual function scores than cases with unilateral NS [22]. In a retrospective study of RARP conducted at nine high-volume centers in Japan from September 2012 to August 2021, 2801 cases of RARP were analyzed, of which 2065 patients (73.7%) were treated by non-NS RARP. Unilateral side NS was performed in 600 patients (21.4%), and bilateral NS was performed in 136 patients (4.9%). Based on this data, bilateral-NS RARP is still rare in Japan [23]. It is highly likely that RARP without NS- or with unilateral NS-RARP is still being performed at many facilities in Japan, so we consider our data valuable. There is a possibility that bilateral RARP will increase from now on, so we would like to accumulate experience in RARP with bilateral NS cases and analyze the comparison with CIRT. Second, we used propensity score matching to analyze data from two cohorts, generating two comparable groups with matched background characteristics. However, this strategy is associated with bias. Future studies should prospectively randomize participants to comparison groups. Third, there were significant differences in baseline pathological characteristics between the groups before matching. The RARP group had a higher Gleason score. Although adjusting for differences in the Gleason score was desirable, we did not match the groups for this score because of the small of number of participants. We considered the impact of the Gleason score to be minor and did not include it among the matched factors. Fourth, information on medical confounders (medication status, diabetes, cardiovascular disease, etc.) that may have affected the sexual function of the patients in the analysis was missing. The propensity score matching method may have biased these factors and made them invisible confounders.

## Conclusions

In conclusion, propensity score-matched comparisons revealed a 40% decrease in the sexual summary score in patients after RARP and a 14% decrease in patients after CIRT. These results offer valuable insights for treatment decision-making of Japanese PCa patients, highlighting the potential benefits of this treatment strategy.

## Data Availability

The datasets used and/or analyzed during the current study are available from the corresponding author on reasonable request.

## Abbreviations

ADT: androgen deprivation therapy
ANOVA: analysis of variance
AS: Active surveillance
CIRT: Carbon-ion radiation therapy
EPIC: Expanded Prostate Cancer Index Composite
GS: Gleason score
IRB: Institutional Review Board
NS: nerve-sparing
PCa: Prostate cancer
PSA: prostate-specific antigen
RCT: randomized controlled trials
QOL: quality-of-life
RARP: robot-assisted radical prostatectomy
RBE: relative biological effect
UCLA-PI: University of California Los Angeles Prostate Cancer Index
